# Role of health extension workers in the relationship between vector control interventions and malaria in Ethiopia

**DOI:** 10.1186/s12879-021-06040-8

**Published:** 2021-11-08

**Authors:** Mohammed Aliye, Tao Hong

**Affiliations:** 1 School of Management, Harbin Institute of Technology, 150001 Harbin, P.R. China; 2 Harbin Institute of Technology, Harbin, Heilongjiang province China

**Keywords:** Malaria prevalence, Strategic interventions, Health extension workers, Structural equation modeling

## Abstract

**Background:**

Despite a tremendous decline in the burden of malaria through public health interventions, it is yet remains a critical parasitic health problem in Ethiopia. Insecticide-treated nets and indoor residual spray are considered as the most effective preventive interventions against malaria. This study intended to determine the role of health extension workers in influencing the relationship between vector control strategies and malaria prevalence in Ethiopia.

**Methods:**

The study adopted a descriptive study based on panel data collected from 10 regions of Ethiopia from 2010 to 2018. The data collected were analyzed using STATA version 13.0. Structural equation modelling was used to assess the mediating effect of health extension workers in the relationship. Further, the random effect model was employed to investigate the direct relationship among the study variables.

**Results:**

We observed a strong mediating role of health extension workers to the relationship between strategic interventions and malaria prevalence, where the direct path is (β = 0.64, *p* < 0.05), and the indirect path (β = 0.72, *p* < 0.001) and (β = 0.98, *p* < 0.001) confirming the mediation condition to appear. Our analysis revealed that, insecticide-treated nets and indoor residual spray significantly impacts the malaria prevalence (β = 0.20, *p* < 0.05) and (β = 0.70, *p* < 0.001) respectively. Further, our analysis suggests that the cumulative effect of indoor residual spray and insecticide-treated mosquito nets have helped better avert malaria prevalence (β = 81.3%, *P* < 0.05). Moreover, the finding demonstrates the incremental rate of 30.2%, which is the indirect effect of the research [(β = 0.813) - (β1 = 0.511)].

**Conclusion:**

The findings are potentially useful for the health sector in charge of infectious disease prevention and control, particularly in developing countries explaining how these group provided support to reduce malaria ensuring the provision of proper health message about the program.

## Background

Malaria is the most critical parasitic health problem in tropical regions [1, 2], a leading cause of morbidity and preventable death [3–6]. Remarkable success is documented in the fight against malaria, especially in Africa [2, 7]. Nevertheless, malaria remains a global health challenge that causes massive morbidity and poses a higher burden of the disease [8]; nearly half of the population of the world lives in the malaria-endemic area [9]. In 2015 the World Health Organization (WHO) estimated that malaria caused 212 million cases worldwide, leading to approximately 429,000 deaths. Africa is the most affected continent with 92% of all malaria deaths mainly occur in children under 5 years [10]. Malaria has been identified as a public health issue in Sub-Saharan Africa, especially in pregnant women and children with the highest risk of severe illness and death [11, 12]. The Ethiopian population has affected by malaria during the planting and harvesting season, causing a decrease in the productive capacity when there is a greater need for work [13, 14]. In 2017, there were more than 1.7 million confirmed malaria cases and recorded as the top ten cause of morbidity among children under 5 years (2.3%) [15]. Likewise, it was the leading cause of outpatient, admission, and death [16]. The Ethiopian health sector has introduced a three-tier health care delivery system, characterized by a Primary Health Care Unit (PHCU); where level one is a Woreda/District health system comprised of a primary hospital (to cover 60,000–100,000 people), health centers (1/15,000–25,000 population) and their satellite Health Posts that serve a kebele comprising (1/3000–5000 population) connected by a referral system. Level two is a General Hospital covering a population of 1–1.5 million people, and level three is a Specialised Hospital covering a population of 3.5–5 million people [17]. PHCU is the lowest level in the Ethiopian health system. A health post is an operational center for two HEWs, which serves 3000–5000 people. A kebele is the smallest administrative unit in Ethiopia, but it can include several small villages. The Ethiopian government has been deploying trained new cadres named health extension workers since 2003, and by the year 2010, a total of 33,819 HEWs were trained and deployed throughout the country, which are required to spend 75% of their time conducting out-reach activities visiting house to house to their respective kebele, while the rest of their time are supposed to be at the health post [18]*.* Many studies investigated vector control strategy (ITNs and IRS) ownership and use for malaria prevention [19–26], and have suggested possible benefits attributable to malaria control, such as the association of ITNs use with malaria knowledge and connection of malaria prevalence with malaria control interventions. Most of these studies relying on cross-sectional data of the specific communities. Moreover, community health workers have identified enhancing the preventive capacity and improve provider-patient linkage [27–30].

Nonetheless, none of the studies recognized the contribution of health extension workers to the relationship between vector control interventions and malaria prevalence. Besides, the previous research fails to add health workers to contribute to the malaria programme in particular. For Ethiopia, ITNS and IRS were hampered by knowledge gaps about malaria transmission, low utilization, and misunderstandings. Therefore, this study aimed at investigating the mediating role of unstudied health extension workers to the improvement of the relationships between ITNs, IRS, and malaria prevalence by employing Structural Equation Modelling (SEM) and by using the period from 2010 to 2018. The study also uses the Random Effect Model (REM) to assess the direct effect of ITNs and IRS on malaria. Also, the study is designed to provide concentrate and adequate evidence to advise malaria prevention and control in this area.

### Health extension workers and malaria

Indications that health extension workers positively impact the relationship between intervention approach and malaria, connected to the preventive characteristics of malaria disease. In Ethiopia, 68% of the landmass is a malarious area, and about 60% of the population is at risk of malaria infection [14, 31]. Insecticide-treated mosquito nets (ITNs) and indoor residual spraying (IRS) were identified as a key approach because of the effectiveness of malaria prevention [32]. Ethiopia is the first country to adopt the scale-up for impact concept (SUFI) for malaria control, and about 50 million insecticide-treated mosquito nets distributed since 2005, and indoor residual spraying scaled up into targeted areas [33]. However, variations on intervention coverage at different sites and problems related to ownership and use of ITNs were reported [34, 35]. Despite the reduction in global malaria cases and deaths [7, 36], challenges with the community’s awareness about net care, use, and repair were reported [37]. Community participation and a responsive approach to malaria control have reported increasing intervention take-up [38]. Community health workers are recognized as an integral part of primary health-care teams, especially in poor and underserved populations [39]. World health organization (WHO) launched “Treat, Train, and retain,” coordinated global effort to address the shortage of health workers across different disease programs, particularly in low-and-middle-income countries [40]. Health workers are defined to be all people engaged in actions whose primary intent is to enhance health, such as physicians, nurses, midwives, public health professionals, community health workers, and all other support workers [41, 42]. For infectious disease prevention and control to succeed, community participation is vital, where vulnerable groups share similar goals. The provision of training skills for community health workers has been reported affecting the transfer of information and treatment of malaria to their respective communities [43]. WHO recognized the creation of capable, motivated, and supported health workers achieving national and global health goals and addressing bottlenecks [44]. Furthermore, community health workers give their clients a way to prevent illness and treat chronic disease [45].

In 2003, Ethiopia initiated the rapid expansion of primary health coverage through a comprehensive health extension program in response to the country’s health problems like malaria. Thus, 10th-grade female high-school graduates were recruited from the area who trained for a full year and returned to the community to promote health and provide village-level services [46]. Two HEWs partnered to represent between 3000 to 5000 people at the health-post level, where they devote much of their time to home visits and outreach. The primary health coverage mostly includes the prevention and control of infectious disease, which is prevented and controlled if accessible intervention tools are utilized by the community. So, the role of health extension workers is to work closely in the society about health promotion and disease prevention, creating awareness on the vulnerability and severity of the disease—the following four hypotheses derived on the basis of the above logical arguments and literature discussions.

**Fig. 1:**
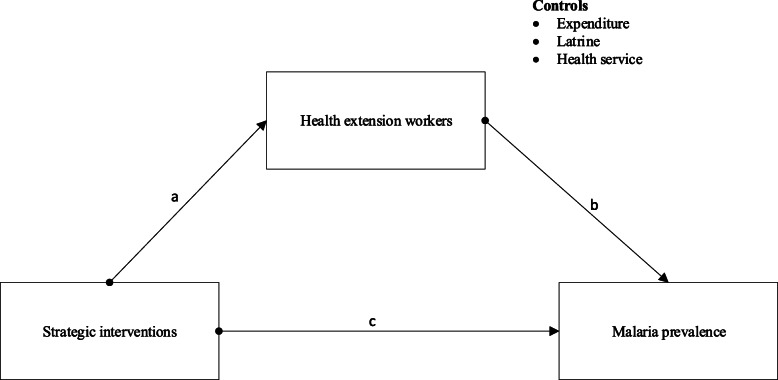
Research model

- H1: Strategic intervention is significantly related to malaria prevalence.
- H2: Strategic intervention is positively related to health extension workers.
- H3: Health extension workers are positively associated with malaria prevalence.
- H4: Health extension workers mediate the relationship between intervention strategy and malaria prevalence (Fig. 1).

## Methods

### Study design, data collection, and study variables

Administratively Ethiopia is structured into nine regional states —Tigray, Affar, Amhara, Oromiya, Somali, Benishangul-gumuz, Southern Nations Nationalities and Peoples (SNNP), Gambela, and Harari and two city administrations, Addis Ababa and Dire Dawa [47]. Thus, a descriptive study based on panel data of 10 endemic entities excluding Addis Ababa was adopted to investigate the relationship between vector control interventions (IRS, ITNs) and malaria prevalence as health extension workers play a mediating role, using national-level data from health and health-related indicators (HHI), fielded annually from 2010 to 2018. The hypotheses above were tested using data collected by the Ethiopian Federal Ministry of Health, in collaboration with the regional health bureau and other stakeholders. HHI includes detailed descriptions of variables related to the malaria program, primarily the prevalence of malaria, deaths of malaria, health workforce, health service expansion, health expenditure, and vector control interventions [47]. After the relevant variables were identified for the current study, we condensed our data as suitable, containing 90 observations.

### Analysis

Social science statistical package, STATA Version 13.0, was used to analyze the data. We followed a two-step approach to conduct the analysis. The paths of hypotheses between constructs were first estimated using the structural equation model (SEM) followed by random effect model (RAM) testing.

### Model testing

All of the hypothesized relationships were examined using structural equation modeling (SEM). SEM method is favored in the causal modeling method as it allows measurement error control, provides information on the degree of fit of the examined model [48]. We tested the theoretical framework in two steps. First, the theoretical framework was examined through the proposed linear relationships among the study variables as well as the mediating effect (hypotheses 1–4) at once in the same model (model 1). Second, the random-effect model (REM) is used to check the direct influence of independent variables on the outcome variable in line with controlling factors (model 2), where REM has the advantage of higher efficiency compared to the FEM resulting in smaller standard errors of coefficients. And high statistical power to detecting effects [49].

Various models were developed to test the direct, indirect, and mediating effect of the study variables. Equation (1) was designed to test the direct influence of indoor residual spraying and insecticide-treated mosquito nets on malaria disease. Equation (2) is designed to test the relationship between health extension workers and vector control measures. Equation (3) for testing the effect of health extension workers on the outcome variable. Equation (4) is used to prove the influence of independent and mediating factors on the outcome of malaria. Eq. (5a&b) and (6) proves the indirect impact of the factors on the relationship. The model uses the log of all variables to minimize data variances. The principal component analysis is also used to capture multiple indicators of our dependent variables and health service expansion. Thus, various models presented as follows:

1 $$ y={\beta}_0+{\beta}_1X+\varepsilon $$

2 $$ M={\beta}_0+{\beta}_1X+\varepsilon $$

3 $$ Y={\beta}_0+{\beta}_1M+\varepsilon $$

4 $$ Y={\beta}_0+{\beta}_1X+{\beta}_2M+\varepsilon $$

To test the indirect effect of the factor in our study, the following two models derived from the above main study equations as follows:

5a $$ \mathrm{Model}\ 1.Y={\beta}_0+{\beta}_1X+{\beta}_0M+\varepsilon $$

5b $$ \mathrm{Model}\ 2.Y={\beta}_0+\beta X+\varepsilon $$

Then, by subtracting β1 from β, we estimate the indirect effect given by:

6 $$ {\beta}_{indirect}=\beta -{\beta}_1 $$

Where Y represents a dependent variable (malaria prevalence), determined as confirmed malaria cases for both Plasmodium species in all age groups [31, 50, 51], X is an independent variable (strategic interventions), calculated for all regions during the 9 years accordingly, and M represents a mediating variable calculated for all study regions, health service expansion, health expenditure, and latrine coverage as a control variable during the study period. While ʎ is the mean, the time-invariant component of those variables [52], β is an estimate of the within effect, and e is the error term. All variables and correlations were found to be significant if *p* < 0.05.

## Results

### Descriptive analysis

The author has empirically investigated 90 observations from 10 malaria epidemic regions of Ethiopia. Data in Table 1 shows descriptive details, i.e., the means (M), standard deviations (SD), and correlations among the study variables. Correlation analysis showed that all of the study variables were positively and significantly associated with each other. All of the associations between the study variables were in the expected directions.

**Table 1:** Descriptive statistics of the research variable

	M	SD	Malaria	Intervention	HEWs	HE	LC	HSE
*MP*	*9.667052*	*2.739*	*1.0000*					
*Interventions*	*11.39936*	*0.536*	0.4910*	*1.0000*				
*HEWs*	*3*	*1.000*	0.6112*	0.5311*	*1.0000*			
*HE*	*6.911924*	*1.502*	0.5827*	0.4507*	0.8107*	*1.0000*		
*LC*	*11.90568*	*2.932*	0.4229*	0.4154*	0.7531*	0.5529*	*1.0000*	
*HSE*	*2.000001*	*1.000*	0.5803*	0.4186*	0.8497*	0.8282*	0.7546*	*1.0000*

Note: *MP* represents malaria prevalence; *HEWs* represents health extension workers; *HE* represents health expenditure; *HSE* represents health service expansion; *LC* represents latrine coverage. *M* Mean, *SD* standard deviation, *p < 0.05

### Hypothesis results

The first step involved addressing the effect of the strategic intervention on malaria prevalence and health extension workers. We checked four steps involved in the Baron and Kenny approach for the establishment of mediation [53]. To establish mediation, 1) the relationship between independent and outcome variable should be significant; 2) significant relationship between independent variable to the hypothesized mediating variable; 3) the mediating variable should be significantly related to the dependent variable, where both the independent and mediating variable is predictors of the dependent variable; 4) a positive coefficient anticipated for the indirect impact of the study, and also a larger coefficient relating independent variable to dependent variable anticipated. The proposed direct and indirect relationships (hypothesis 1–4) were simultaneously tested in the structural equation model. The results are depicted in Table 2 (model 1). Consistent with the researcher’s anticipation of a positive relationship between strategic intervention and malaria prevalence, the path was found to be significant (β = 0.64, *p* < 0.05). It was concluded that strategic intervention has a direct positive effect on the prevalence of malaria; thus, H1 and condition 1 was accepted. The researchers also attempted to determine whether a strategic intervention has a positive influence on health extension workers. As proposed, the path coefficient was positive (β = 0.98) and statistically significant (*p* < 0.000), supporting the H2 notion and meeting condition 2. Moreover, health extension workers had a significant effect on malaria prevalence (β = 0.72, *p* < 0.001), supporting hypothesis 3, fulfilling condition 3. Finally, the researcher addresses the mediating effect of health extension workers in line with the direct impact, where path a + b is the indirect effect, and path c is the direct effect. Consistent with the study expectation of a positive relationship between independent and dependent variables via mediator, the path was found to be significant, where path a (β = 0.98, *p* < 0.001), path b (β = 0.72.3, *p* < 0.001) and path c (β = 0.64, *p* < 0.05). Thus, hypothesis 4 regarding the mediation effect was supported. Likewise, a positive coefficient for the indirect path (β = 0.30), and a larger coefficient of independent to dependent relationship existed (β = 0.81, *p* < 0.05), (β1 = 0.51, *p* < 0.05) meeting the 4th mediation analysis approach. The study also shows the significant role of health extension workers in the relationship between strategic interventions and malaria prevalence with 74% (0.98 + 0.43–0.67) if mediation effect in place, and 0.67 without mediation.

**Table 2:** Results of Structural Equation Model Path Analysis

	Path	Coefficient	SE	Z	P	[95% Conf. Interval]	
*Controls*							
Malaria prevalence <−	*Health expenditure*	*.2065569*	*.2951408*	*0.70*	*0.484*	*−.3719084*	*.7850223*
Malaria prevalence <−	Laterine coverage	*−.1371008*	*.1269505*	*−1.08*	*0.280*	*−.3859193*	*.1117176*
Malaria prevalence <−	H/S/expansion	.6890804	.5170457	1.33	*0.183*	−.3243106	1.702471
*Main Effects*							
Malaria prevalence <−	*Interventions*	*.6350601*	*.261278*	*2.43*	*0.015*	*.1229646*	*1.147156*
Malaria prevalence <−	HEWs	*.722892*	*.1412621*	*5.12*	*0.000*	*.4460233*	*.9997607*
HEWs <−	Interventions	.9822345	.165201	5.95	*0.000*	.6584465	1.306023

Note: *HEWs* denotes health extension workers

### Results of the direct relationship

Table 3 presents a detailed description of associations between research variables. Thus, the results of a random effect model regressions offered to show the relationship between dependent, mediating, and independent variables. The effect of control variables on the outcome of malaria was also presented. The model provides an assessment of vector control methods (IRS & ITNs) and health extension workers to impact malaria prevalence. The result showed a positive relationship between strategic intervention and malaria prevalence (β = 81.3, *p* < 0.01), which indicates strategic mechanisms conquer malaria control at model 1. A significant relationship reported on the impact of intervention on malaria prevalence revealing the attribution of the strategy to prevent malaria (β = 0.511, *p* < 0.05, and β = 0.487, *p* < 0.05) as shown in model 2 and 3 respectively. Moreover, the study demonstrates the positive association of controlling factors such as health service expansion and health expenditure to malaria prevalence, with a significant value at 0.1. Further, a negative relationship was reported for latrine coverage, revealing an indirect effect of this factor on malaria prevention actions.— Supported by the report that showed the indirect effect of latrine coverage on the malaria program due to the reduction in the accumulation of dust around households, which paves the way for reduced malaria transmission [54]. Moreover, the research revealed differences in the attribution of malaria prevention when two independent factors of vector control measures were implemented. The study discovered strong relationship between indoor residual spray to the outcome variable than the insecticide-treated mosquito net does (β = 0.70, *p* < 0.001) and (βn = 0.20, *p* < 0.05) respectively. The variance among principal vector control methods observed maybe because the indoor residual spray is a one-time action in a year, while ITNs is a daily activity that needs the community understanding of susceptibility and severity of the disease to take action. Thus, the study demonstrates the cumulative effect of intervention strategy better attribution in malaria prevention and control than stand alone.

**Table 3:** Result of the direct relationship

	(1)	(2)	(3)	(4)	(5)
	Malaria prevalence	Health extension workers	Malaria prevalence	Malaria prevalence	Malaria prevalence
Interventions	0.813^**^	−0.0490		0.511^*^	0.487^*^
	(3.16)	(−0.93)		(2.03)	(2.04)
Health extension workers			0.872^***^	0.755^***^	0.220
			(4.16)	(3.71)	(0.70)
Health Expenditure					0.497
					(1.71)
Latrine coverage					−0.208
					(−1.67)
Health service expansion					1.016
					(1.74)
constant	7.229^***^	7.059^***^	3.639^*^	2.918^*^	−2.714
	(8.38)	(18.72)	(2.42)	(2.04)	(−0.53)
*N*	90	90	90	90	90

Note: N represents number of observations; *t* statistics in parentheses; ^*^ *p* < 0.05, ^**^ *p* < 0.01, ^***^ *p* < 0.001

## Discussion

Low and middle-income countries are characterized by healthcare systems that are already fragile due to weak infrastructures, a scarcity of health workers, and limited financial resources. Malaria is yet a huge killer, representing the biggest health problem particularly in the poverty context globally. For countries with limited human and financial resources, such as Africa, prevention strategies are the utmost potential [55], which can be achieved through educating people about the program on prevention and control actions through trained professionals sustainably. In the era of pandemic diseases such as Ebola, SARS, and COVID, it is pertinent to underline how health extension workers impact to improve the relationship where they may have similar aspects and seem to have a strong potential for mutual influence. In this regard, strengthening the prevention actions aligned with the cadres of the program is critical.

Thus, the goal of the present research was to examine the interplay of strategic interventions, health extension workers, and malaria in the context of the health program. Besides, the study investigates the cumulative effect of IRS and ITNS (strategic interventions) to protect malaria. This study showed a significant relationship between IRS, ITNs, and malaria prevalence. Similar findings have also been reported a reduction in the outcome of malaria cases and malaria related-deaths due to IRS and ITNs implementation [7, 36], effective protection of ITNs use also reported [56]. Further, for malaria control, the present study proved a positive and better attribution of strategic interventions to fight against malaria. This finding is consistent with results in other studies from different malaria-endemic regions around the world, which indicates significant added protection from a combination of IRS and ITNs [26, 57].

Moreover, the study showed a positive relationship between strategic intervention and health extension workers, and health extension workers were also positively related to malaria prevalence. Furthermore, this study reported that health extension workers mediated the relationship between strategic intervention and malaria prevalence. This argument is consistent with the study which reported community health workers to improve performance efficiency [27, 58]. Furthermore, the direct and indirect relationship through health extension workers between strategic interventions and malaria prevalence was stronger. Our result corroborated with the study finding, which reported that community health workers give their clients a way to prevent illness and treat chronic disease [45]. Hence the primary constraint, particularly in developing countries, is a severe shortage of health workers; the role of community health workers is unmeasurable in disease prevention. A positive impact of community workers on access and coverage outcomes of HIV patients was also reported [59]. The results of the current study show the tremendous impact of health extension workers in strengthening the relationship between strategic interventions and malaria disease and contributed to malaria prevention and control programs. However, a negative relationship between IRS, ITN and malaria was reported showing a small number of malaria cases before the implementation of these intervention [60]. The study reported the role of community health workers in building the capacity of the society and promoting patient empowerment and the entire spectrum of prevention levels [29]. There are numerous reasons why the described program has an impact on increasing the community’s role in malaria control. Health extension workers can play a role in the spread of professional messages on communicable disease prevention, such as malaria, and in the outcome of infection. The program also includes the elements that have been associated with the ownership and utilization of health interventions. Thus, the present study described novel involvements for ITNs and IRS impact on malaria protection through the connection of health extension workers as mediating roles. In agreement with the role of health extension workers to influence protective ability, the community health workforce has been reported as effective to enhance health literacy and provider-patient communication [61]. Possible attribution of health extension workers in improving maternal health service also suggested [62], however, problems on health service delivery and skilled birth attendance were cited.

## Conclusion

A high burden imposed from malaria on endemic regions of Ethiopia, where it accounts for the number of mortality and morbidity, mostly in pregnant women and children. However, most studies do not provide evidence on the role of health extension workers in malaria prevention and control. We provide rigorous evidence on the effects of health extension workers in improving the relationship between intervention strategy and the malaria disease. Previous research has shown the role of the health workforce in improving health outcomes. This study typically explores the specific effect of health extension workers that can act to these relationships towards prevention and control of the malaria disease. Employing structural equation modeling and the random effect model, we found a positive and incremental impact of vector control interventions on malaria. Essential questions also left that pave the way for future researchers to address, among which, providing tangible evidence on the implementation of the program, which can be solved through observation and focus group discussion with service providers and the community. It would also be of great importance to discover the cumulative effect of the total health workforce for disease protection. Also, to note the differences in the health outcome to identify the focus area to which health sector actors give attention to infectious disease prevention. Besides, we are missing information on how health extension workers run the program in society and program challenges on the implementation.

### Implication

This study that has presented should be of particular interest to the organization operating in the health sector, particularly in developing countries, which are vulnerable to infectious disease. Sub-Saharan African countries are mostly experiencing the most significant public health threats, which can be preventable if appropriately addressed. The study demonstrates the provision of proper health education via health extension workers, also of potential interest for the nations which face the highest challenges of health workforce shortages. The WHO report shows the experience of a chronic deficiency of a well-trained health workforce in the world. There are 57 countries, mostly in sub-Saharan Africa, but also including Bangladesh, India, and Indonesia, that face crippling health workforce shortage [41]. To the best of knowledge, no empirical evidence available on the relationship between strategic intervention approaches and malaria as health extension workers as a mediating role. Our study finding represents the initial step towards filling this gap. The reason behind the researchers to these relationships is to give insights for policymakers to give due focus on health extension workers’ effect on these relationships. Also, align with intervention provision to assure the suitable achievement to the intended goals. The generalization of the study finding and implications is not limited to the context of malaria. Rather social workers dealing with the well-being of the health of society, particularly infectious diseases, also be subject to the mechanisms described.

## Data Availability

Health and health-related indicators data from 2010 to 2018 are available at: (http://ghdx.healthdata.org/record/ethiopia-health-and-health-related-indicators-2010-2011, http://ghdx.healthdata.org/record/ethiopia-health-and-health-elated-indicators-2011-2012, http://ghdx.healthdata.org/record/ethiopia-health-and-health-related-indicators-2012-2013, http://ghdx.healthdata.org/record/ethiopia-health-and-health-related-indicators-2013-2014, http://ghdx.healthdata.org/record/ethiopia-health-and-health-related-indicators-2014-2015, http://ghdx.healthdata.org/record/ethiopia-health-and-health-related-indicators-2015-2016, http://repository.iifphc.org/bitstream/handle/123456789/395/Health%20and%20Health%20Related%20Indicator%202017.pdf?sequence=1&isAllowed=y, http://repository.iifphc.org/bitstream/handle/123456789/395/Health%20and%20Health%20Related%20Indicator%202018.pdf?sequence=1&isAllowed=y) all data are freely accessible. The compiled data upon which the result based could be accessed at a reasonable request.

## Abbreviations

ITNs: Insecticide-treated nets
IRS: Indoor residual spray
MoH: Ministry of Health
PMI: Presidents Malaria Initiatives
RDT: Rapid Diagnostic Test
WHO: World Health Organization
HHI: Health & Health-Related Indicators
HMIS: Health Management Information System
HEWs: Health Extension Workers
SEM: Structural Equation Modelling
REM: Random Effect Model
